# Severe Anaplasmosis with Multi-Organ Failure in a Patient with Splenectomy: A Case Report

**DOI:** 10.3390/idr17020038

**Published:** 2025-04-21

**Authors:** Nithin Karnan, Predrag Jancic, Igor Dumic, Emeka Amadi, Vishnu Kommineni, Jelena Stojsavljevic, Aryan Shiari, Melissa Hart, Ra’ed Jabr, Charles W. Nordstrom

**Affiliations:** 1 Department of Hospital Medicine, Mayo Clinic Health System, Eau Claire, WI 54703, USA; nithinkarnan@gmail.com (N.K.); prjancic@gmail.com (P.J.); amadi.emeka@mayo.edu (E.A.); kommineni.vishnuteja@mayo.edu (V.K.); stojsavljevic.jelena@mayo.edu (J.S.); nordstrom.cw@mayo.edu (C.W.N.); 2 Mayo Clinic College of Medicine and Science, Rochester, MN 55905, USA; shiari.aryan@mayo.edu (A.S.); hart.melissa3@mayo.edu (M.H.); jabr.raed@mayo.edu (R.J.); 3 Department of Pulmonary and Critical Care Medicine, Mayo Clinic Health System, Eau Claire, WI 54703, USA; 4 Department of Pathology, Mayo Clinic Health System, Eau Claire, WI 54703, USA; 5 Department of Infectious Disease, Mayo Clinic Health System, Eau Claire, WI 54703, USA

**Keywords:** anaplasma, tick-borne, multiorgan failure, splenectomy, acute renal failure, rhabdomyolysis, myocarditis, respiratory failure, atrial fibrillation

## Abstract

Background: *Anaplasma phagocytophilum* is an emerging tick-borne zoonotic pathogen that typically causes mild infections, which are often successfully managed in outpatient settings. Immunosuppression associated with splenectomy is a well-documented risk factor for severe infections from pathogens such as *Babesia microti* and encapsulated bacteria. However, splenectomy has not previously been identified as a risk factor for severe anaplasmosis. Case Presentation: This report describes a rare case of severe anaplasmosis complicated by multiorgan failure in a patient who had undergone splenectomy several decades earlier. The clinical course was notable for pneumonia, acute respiratory distress syndrome, acute kidney injury, rhabdomyolysis, atrial fibrillation, and possible myocarditis. Despite the severity of the presentation, prompt initiation of doxycycline led to recovery, albeit with a significantly prolonged hospital stay. Conclusions: Patients with splenectomy might be more likely to develop a serious form of *Anaplasmosis* infection such as multiorgan failure. Clinicians in tick-borne endemic areas should be aware that non-specific symptoms can indicate anaplasmosis.

## 1. Background

*Anaplasma phagocytophilum* is an intracellular obligate Gram-negative bacterium, the causative agent of human granulocytic anaplasmosis (HGA). In the United States this disease is endemic in the Northeast, upper Midwest, and Pacific Northwest [1]. The annual incidence of anaplasmosis in the United States is approximately 5000 cases [2], and within the United States infection is transmitted by two species of blacklegged tick, *Ixodes scapularis* and *Ixodes pacificus*. Infection can rarely be acquired from exposure to bodily fluids and by blood transfusion [3,4].

The clinical presentation of anaplasmosis varies widely, ranging from asymptomatic infection to severe illness. The most common symptoms include fever and constitutional complaints, followed by gastrointestinal (GI) manifestations such as nausea, vomiting, and abdominal pain [5]. Myalgia, headache, and rash are also frequently observed. In rare cases, HGA can cause severe illness, leading to encephalitis [6], pneumonia [7,8], myocarditis [9], and hematophagocytic lymphohistiocytosis (HLH) [10,11].

## 2. Case Presentation

An 83-year-old woman with a history of recurrent idiopathic pancreatitis, previously treated with partial pancreatectomy and splenectomy (40 years prior), presented with a one-day history of fever, shortness of breath, nausea, and vomiting. She had been in her usual state of health prior to the onset of these symptoms, with no known recent sick contacts, or travel outside of Wisconsin, USA, within the past year. The patient denied any history of alcohol use, smoking, or illicit drug use. She hiked regularly, was an avid gardener, and she had a dog but denied any dog bites or tick bites.

Upon admission, the patient was normotensive but tachypneic, requiring 2 L of oxygen to maintain her oxygen saturation above 92%. Chest radiography demonstrated an opacity in the right lung. Initial laboratory evaluation revealed significantly elevated liver function tests, hyponatremia, and an elevated lactate level, while the complete blood cell (CBC) count was within normal limits. Blood cultures were obtained, and the multiplex respiratory viral panel was negative. A summary of comprehensive tests performed during the hospitalization is provided in Table 1.

**Figure 1:**
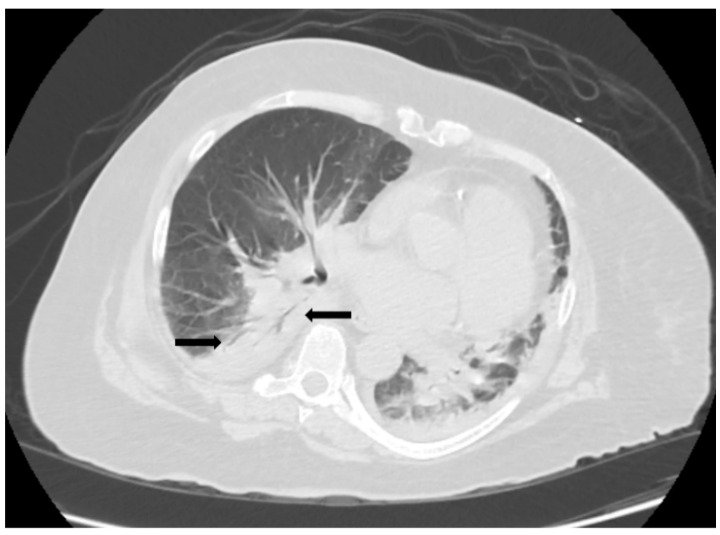
Computed Tomography (CT) of the thorax showing bilateral lower lobe areas of airspace opacity with air bronchogram sign (arrows).

The patient was initiated on ceftriaxone and azithromycin, along with intravenous fluids, for suspected community-acquired pneumonia (CAP). However, her condition worsened overnight, marked by the onset of wheezing and worsening hypoxia, necessitating non-invasive ventilatory support. Intravenous steroids were added to her regimen to address reactive airway disease and severe CAP. A non-contrast CT of the chest demonstrated bilateral lower lobe consolidation with air bronchograms, centrilobular ground-glass opacities in the upper lobes consistent with pneumonia, and trace bilateral pleural effusions (Figure 1).

**Figure 2:**
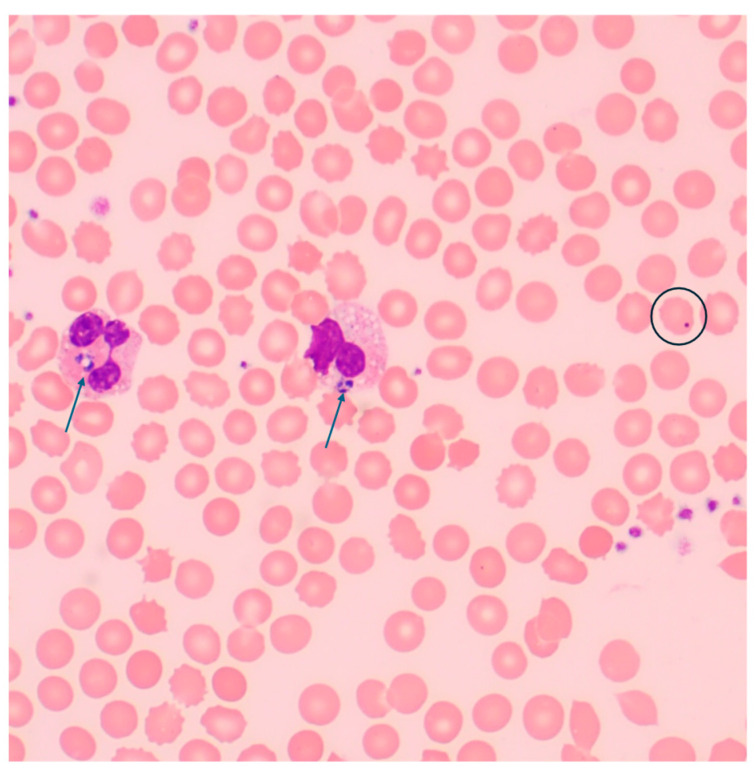
Peripheral blood smear on 50× objective (500× magnification). On microscopic examination of the peripheral smear, numerous neutrophils contained cytoplasmic inclusions consistent with anaplasmosis (blue arrows). Background red cells demonstrated features of asplenism including Howell-Jolly bodies (black circle).

On the second day of hospitalization, a peripheral blood smear revealed atypical cells containing cytoplasmic inclusions within the neutrophils (Figure 2), and the findings were highly suggestive of anaplasmosis. This diagnosis was subsequently confirmed by a positive blood PCR test conducted at Mayo Clinic Medical Laboratories.

Azithromycin was discontinued, and the patient was transitioned to intravenous doxycycline. In addition to respiratory failure, her clinical course was complicated by acute kidney injury, rhabdomyolysis, and anion gap metabolic acidosis. Intravenous hydration was continued, and targeted interventions were implemented to correct the hyponatremia and metabolic acidosis. The trend of pertinent laboratory values is summarized in Table 2.

**Figure 3:**
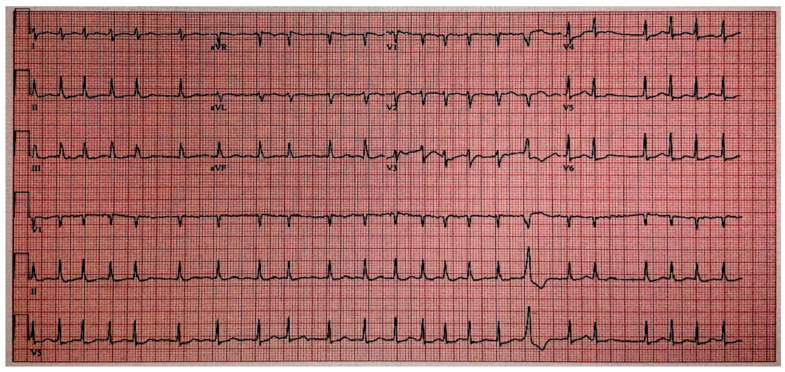
Electrocardiogram showing the presence of an irregular rhythm and absence of P-waves.

On the third day of hospitalization, the patient developed atrial fibrillation (Figure 3) with rapid ventricular response.

Anaplasmosis-related myocarditis was suspected, prompting a transthoracic echocardiogram, which demonstrated a normal ejection fraction without regional wall motion abnormalities, and no pericardial effusion. A rate-control strategy was implemented utilizing a beta-blocker and heparin infusion.

The following day, despite correction of hyponatremia and metabolic acidosis, the patient developed encephalopathy, characterized by lethargy, decreased responsiveness, and confusion. Physical examination revealed no meningeal signs, and lumbar puncture was deferred due to anticoagulation therapy. The following day, her mental status began to improve, and by hospital day 6, she had returned to her baseline cognitive function. The encephalopathy was ultimately attributed to hospital-acquired delirium due to critical illness.

Given the patient’s significantly elevated ferritin levels and febrile pancytopenia, hemophagocytic lymphohistiocytosis (HLH) was considered. A cytokine panel performed during her hospitalization revealed markedly elevated levels of IL-2 soluble receptor alpha, IL-18, and TNF, supporting the suspicion of HLH. However, as her clinical condition and laboratory markers improved with intravenous methylprednisolone and doxycycline, further invasive testing was deemed unnecessary.

The patient’s clinical condition improved, and she was discharged on hospital day 12 with a 2-week course of oral doxycycline to complete her treatment. Despite this improvement, she continued to experience significant weakness and difficulty performing daily activities, necessitating transfer to a skilled nursing facility for continued rehabilitation and care.

## 3. Discussion and Conclusions

In immunocompetent individuals, anaplasmosis is typically a mild infection that can be successfully treated in an outpatient setting. Severe organ failure and cytopenias necessitating hospitalization and close monitoring are rare. Multi-organ failure (MOF) associated with anaplasmosis is uncommon; however, it has been reported in the literature [12,13,14,15]. For example, one case described fatal arrhythmia leading to anoxic brain injury, and death [13]. Another case highlighted the life-threatening nature of transfusion-transmitted anaplasmosis, where a patient developed MOF following surgery and ultimately succumbed to the illness despite receiving appropriate therapy immediately after diagnosis [14]. In this instance, mortality was attributed to diagnostic delay, as anaplasmosis is not typically suspected as a cause of febrile pancytopenia in the postoperative period. Furthermore, patients requiring transfusions are often chronically ill and/or immunosuppressed, factors that increase the mortality risk in this population [14]. In another report, nonspecific initial symptoms of anaplasmosis went unrecognized, and delayed diagnosis and treatment resulted in the patient’s death from MOF [15].

During her hospital stay, our patient developed lethargy, raising concerns for potential encephalitis. However, her encephalopathy was ultimately attributed to delirium in the context of critical illness. Although *Anaplasma phagocytophilum* is not known to directly affect neurons, the pathogen can occasionally be detected in the cerebrospinal fluid (CSF). The neurological manifestations are thought to result from cytokine activation rather than direct neuronal injury caused by the bacterium [6].

Although rare, anaplasmosis should be considered as a potential cause of community acquired pneumonia (CAP) in patients from endemic areas who present with dyspnea, fever, cough, and laboratory evidence of cytopenia and/or liver enzyme abnormalities [7,8]. Empiric antibiotic therapy with doxycycline is recommended in such cases while awaiting the results of diagnostic confirmation, as commonly used antibiotics for CAP, such as ceftriaxone, levofloxacin, and azithromycin, are ineffective against anaplasmosis and may allow progression of pneumonia and worsening hypoxic respiratory failure [8]. Radiological findings in patients with pneumonia caused by *Anaplasma phagocytophilum* include bilateral ground-glass opacities, infiltrates, and/or bilateral pleural effusion [8]. Notably, given the low index of suspicion for anaplasmosis pneumonia, initial empirical antimicrobial regimens failed to include doxycycline in approximately 80% of reported cases [8].

Acute respiratory distress syndrome (ARDS) is an uncommon complication of anaplasmosis, but has been reported, particularly in cases where treatment was delayed [8,9,10,11,12]. ARDS is characterized by acute onset within one week of a known clinical insult, with bilateral opacities on chest imaging not fully explained by effusions, congestive heart failure, or fluid overload. The severity of ARDS is classified based on the PaO_2_/FiO_2_ ratio, with specific thresholds for mild, moderate, and severe cases [16,17]. In our case, the patient was admitted with respiratory failure and hypoxia, with imaging and laboratory findings consistent with ARDS. Fortunately, prompt initiation of antimicrobial therapy targeting anaplasmosis resulted in significant improvement in her hypoxia, ultimately leading to the resolution of ARDS and full recovery of respiratory function by discharge.

Splenectomy is a well-recognized risk factor for severe infections caused by encapsulated bacteria, such as *Streptococcus pneumoniae*, and for parasite *Babesia microti*, which share the same tick vector as *Anaplasma phagocytophilum*. The increased risk and severity of infections following splenectomy are attributed to impaired immunoglobulin production, inadequate opsonizing filtration by the spleen, and the absence of splenic macrophages [18]. Functional hyposplenism, such as that observed in sickle cell disease, can similarly predispose patients to disseminated infections due to the inability of splenic macrophages to clear infected cells [13]. Post-splenectomy sepsis typically presents as a flu-like prodrome followed by rapid progression to septic shock within 48 h of disease onset. Despite appropriate treatment, mortality remains high. While the risk of infection is greatest within the first three years following splenectomy, it persists for life, as evidenced by reports of fulminant infection occurring decades later [13]. Preventative strategies, including patient education, vaccination, and, in select cases, antibiotic prophylaxis, are critical in reducing infection risk. Equally important is maintaining a high index of suspicion for infections in this population and initiating prompt antibiotic therapy when needed. To our knowledge, this is the first documented case of severe anaplasmosis in a post-splenectomy patient, underscoring the importance of maintaining a high index of suspicion for anaplasmosis in endemic areas and the need for early initiation of appropriate therapy.

Our patient presented with atrial fibrillation with rapid ventricular response, likely attributable to a combination of factors, including critical illness, hypoxia secondary to pneumonia, and possibly myocarditis associated with anaplasmosis. Although cardiac involvement in tick-borne diseases has traditionally been associated with Lyme disease in the form of myocarditis and heart block [19], emerging evidence suggests that other tick-borne diseases, such as babesiosis [20] and ehrlichiosis [21] can also impact the heart. Among these tick-borne illnesses, anaplasmosis remains a rare but increasingly recognized cause of myocarditis and arrhythmia [22,23].

Severe anaplasmosis can result in acute kidney injury (AKI) and rhabdomyolysis [24,25,26,27]. Patients may present with gross hematuria, and urinalysis may reveal distorted red blood cells. Kidney biopsy findings in such cases may be consistent with membranoproliferative glomerulonephritis, similar to renal involvement seen in Lyme disease and ehrlichiosis [24]. A recent systematic review of 110 cases of anaplasmosis reported that approximately 15.5% of patients developed AKI, with some requiring continuous renal replacement therapy (CRRT) due to the severity of the injury [5,25]. Rhabdomyolysis may occur via two mechanisms: direct cell membrane destruction or cell energy deficit [26]. It is thought that *A. phagocytophilum* induces renal injury primarily through direct cell membrane damage or indirectly via aberrant immune system activation involving macrophages and cytokine release [28]. However, the exact mechanism remains unclear. Rhabdomyolysis has also been documented in other tick-borne illnesses, both bacterial and viral [29,30] with similar immunologic mechanisms noted in *Borrelia* spp., including cytokine mediated damage, and, in some cases, direct muscle invasion [31]. Similar mechanisms have been hypothesized as a mechanism of injury in Mediterranean spotted fever, a tick-borne illness similar to Rocky Mountain spotted fever, where direct muscle damage may play a role [32].

Our patient’s elevated ferritin level raised concern for secondary hemophagocytic lymphohistiocytosis (HLH) as a complication of anaplasmosis. HLH represents the most severe complication within a spectrum of Anaplasma-associated illnesses [28]. These conditions share overlapping clinical features, including fever, pancytopenia, elevated ferritin and triglyceride levels, and splenomegaly [33]. A key distinction between anaplasmosis mediated HLH and HLH secondary to other causes (autoimmune disorders and malignancies), lies in the rapid response to doxycycline [34,35]. Notably, up to 50% of patients with HLH caused by tick-borne infections improved with timely antimicrobial treatment alone and did not require immunosuppressive therapy [10]. HLH due to tick-borne infections tends to have a better prognosis than HLH caused by other infections or malignancies [10]. In many cases, prompt treatment of the underlying infection eliminates the trigger, facilitating HLH resolution without requiring additional immunosuppressive interventions [10]. Given the extremely high mortality rate of patients with HLH, we suggest that all cases of suspected or confirmed HLH undergo testing for anaplasmosis and other tick-borne infections. If there is a history of possible tick exposure, patients should be empirically treated with doxycycline while awaiting further testing.

Anaplasmosis has the potential to manifest as a severe infection, particularly in patients who have undergone splenectomy, placing them at a heightened risk for multi-organ failure and other severe complications. These patients might not be able to mount an appropriate immune response and may not develop antibodies against the pathogen, which limits the diagnostic yield of antibody testing. Molecular techniques such as PCR are preferred mode of testing for anaplasmosis and have higher sensitivity than antibody testing. Recent review on anaplasmosis described that in 72% of cases diagnosis was achieved by blood PCR testing, while serology and peripheral blood smear were used less commonly [5].

Clinicians practicing in endemic areas must maintain a high index of suspicion for atypical presentations and nonspecific symptoms associated with anaplasmosis. Timely testing by molecular techniques and prompt initiation of doxycycline therapy is critical and typically results in rapid resolution of symptoms and clinical signs. However, further research is essential to elucidate the specific risks of anaplasmosis in splenectomy patients, to define the full spectrum of clinical manifestations, and to determine the optimal duration of treatment. Furthermore, future studies should aim to determine whether prophylactic doxycycline use in splenectomized patients following tick bites can prevent the development of anaplasmosis or mitigate the severity of infection if the disease does occur.

## Figures and Tables

**Table 1:** Comprehensive list of tests performed during hospitalization.

	Specimen			
Test	Blood	Urine	Sputum	Nasopharyngeal Swab
Bacteria/Candida Cultures	Negative		Negative	
*Legionella pneumophila* antigen		Negative		
*Streptococcus pneumoniae* antigen		Negative		
*Staphylococcus aureus*, PCR				Negative
Methicillin resistant *Staphylococcus aureus,* PCR				Negative
Peripheral smear	Intraneutrophilic inclusion			
*Pneumocystis jiroveci* PCR			Negative	
Adenovirus				Negative
Coronavirus 229E				Negative
Coronavirus HKU1				Negative
Coronavirus NL63				Negative
Coronavirus OC43				Negative
Coronavirus-2				Negative
*Influenza A* Virus				Negative
*Influenza B* Virus				Negative
*Respiratory Syncytial Virus*				Negative
*Borrelia burgdorferi* PCR	Negative			
*Ehrlichia chaffeensis* PCR	Negative			
*Ehrlichia ewingii/canis* PCR	Negative			
*Ehrlichia muris eauclairensis* PCR	Negative			
*Babesia microti* PCR	Negative			
*Babesia duncani* PCR	Negative			
*Babesia divergens/MO-1* PCR	Negative			
*Borrelia miyamotoi* PCR	Negative			
Lyme Ab Modified 2-Tier w/Reflex	Negative			
*Anaplasma phagocytophilum* PCR	Positive			
Hepatitis B Surface Antigen Screening	Negative			
Hepatitis C IgM Ab	Negative			
Hepatitis A IgM Ab	Negative			
Hepatitis C virus Ab w/Reflex to Hepatitis C virus PCR, Serum	Negative			
(1,3) Beta-D-Glucan, Quantitative	Negative			
Aspergillus Ag	Negative			
Coccidiosis Ab Screen	Negative			
Histoplasma/Blastomyces Ag Result	Negative			
Cryptococcus Ag Screen w/Titre	Negative			

Abbreviations: PCR—polymerase chain reaction; Ab—antibody; Ag—antigen.

**Table 2:** Serum general chemistry results and complete blood count during the acute illness.

		Day									
	Ref. Range	1	2	3	4	5	6	7	8	9	10
Sodium	135–145 mmol/L	132	**125**	**127**	**131**	**130**	**128**	136	139	138	138
Bicarbonate	22–29 mmol/L	24	26	22	**16**	22	23	27	28	29	29
CPK	26–192 U/L		**2444**	**2491**	**3085**	**1624**	**896**	**819**	**724**		
Creatinine	0.59–1.04 mg/dL	0.74	0.89	**1.98**	**2.63**	**2.61**	**2.15**	**2.13**	**2.16**	**1.95**	**1.76**
GFR	≥60 mL/min/BSA	80	64	**25**	**18**	**18**	**20**	**21**	**22**	**25**	**28**
Bilirubin, Total	0.0–1.2 mg/dL	0.6	1.0	0.5	0.3	0.3	0.3	0.4	0.5		0.9
ALT	7–45 U/L	**282**	**255**	**191**	**165**	**129**	**123**	**110**	**94**		**63**
AST	8–43 U/L	**423**	**334**	**264**	**205**	**125**	**116**	**85**	**65**		39
ALP	35–104 U/L	**185**	**209**	**201**	**208**	**178**	**161**	**141**	**138**		**120**
Troponins	≤10 ng/L	**45**	**183**	**192**	**165**		**134**				
Procalcitonin	0.00–0.24 ng/mL	**0.34**		**4.88**	**56.01**			**8.11**	**3.55**		
CRP	<5.0 mg/L	**128.1**		**272.2**	**164.0**	**69.7**				**11.8**	
Ferritin	11–328 mcg/L		**3338**	**5116**	**1989**	**1235**	**817**	**498**		**361**	
Triglyceride	<150 mg/dL		75			**329**	**136**				
Hemoglobin	11.6–15.0 g/dL	13.9	13.2	13.1	12.6	12.3	**11.5**	**11.3**	**10.9**	**9.0**	**9.2**
Hematocrit	35.5–44.9%	41.0	38.6	38.0	37.3	**34.5**	**32.9**	**32.2**	**32.0**	**27.0**	**27.0**
Platelet count	157–371 × 10^9^/L	248	190	172	**153**	**153**	159	195	277	394	521
Leukocytes	3.4–9.6 × 10^9^/L	8.1	7.6	**10.9**	9.2	**13.0**	**16.8**	**20.6**	**15.4**	**13.7**	**12.1**

Abbreviations: CPK—creatine phosphokinase; GFR—glomerular filtration rate; ALT—alanine transaminase; AST—aspartate transaminase; ALP—alkaline phosphatase; CRP—C-reactive protein. All abnormal values are presented in bold.

## Data Availability

All data are included within the manuscript. This is a case report not a research article.
